# Magnesium-Free Immobilization of DNA Origami Nanostructures at Mica Surfaces for Atomic Force Microscopy

**DOI:** 10.3390/molecules26164798

**Published:** 2021-08-07

**Authors:** Yang Xin, Amir Ardalan Zargariantabrizi, Guido Grundmeier, Adrian Keller

**Affiliations:** Technical and Macromolecular Chemistry, Paderborn University, Warburger Str. 100, 33098 Paderborn, Germany; yangxin@mail.uni-paderborn.de (Y.X.); ardalan@mail.uni-paderborn.de (A.A.Z.); g.grundmeier@tc.uni-paderborn.de (G.G.)

**Keywords:** DNA origami, DNA nanotechnology, adsorption, mica, atomic force microscopy

## Abstract

DNA origami nanostructures (DONs) are promising substrates for the single-molecule investigation of biomolecular reactions and dynamics by in situ atomic force microscopy (AFM). For this, they are typically immobilized on mica substrates by adding millimolar concentrations of Mg^2+^ ions to the sample solution, which enable the adsorption of the negatively charged DONs at the like-charged mica surface. These non-physiological Mg^2+^ concentrations, however, present a serious limitation in such experiments as they may interfere with the reactions and processes under investigation. Therefore, we here evaluate three approaches to efficiently immobilize DONs at mica surfaces under essentially Mg^2+^-free conditions. These approaches rely on the pre-adsorption of different multivalent cations, i.e., Ni^2+^, poly-l-lysine (PLL), and spermidine (Spdn). DON adsorption is studied in phosphate-buffered saline (PBS) and pure water. In general, Ni^2+^ shows the worst performance with heavily deformed DONs. For 2D DON triangles, adsorption at PLL- and in particular Spdn-modified mica may outperform even Mg^2+^-mediated adsorption in terms of surface coverage, depending on the employed solution. For 3D six-helix bundles, less pronounced differences between the individual strategies are observed. Our results provide some general guidance for the immobilization of DONs at mica surfaces under Mg^2+^-free conditions and may aid future in situ AFM studies.

## 1. Introduction

Introduced 15 years ago, DNA origami technology [1,2] has evolved into a popular nanofabrication method that nowadays is almost routinely employed in numerous areas of biomedical research [3,4], single-molecule biochemistry [5,6,7] and biophysics [8,9], bioanalytics and biosensing [10,11], and synthetic biology [12,13], among others. It relies on the controlled folding of a long single-stranded DNA scaffold into a user-defined 2D or 3D nanoscale shape upon hybridization with a large set of short oligonucleotides called staple strands. The shape of the resulting DNA origami nanostructures (DONs) is fully determined by the sequences of the staple strands and can thus be tailored with molecular accuracy to meet the criteria of the desired application. By conjugating chemical moieties to selected staple strands, this technique furthermore enables the controlled arrangement of functional molecules with sub-nanometer precision [14,15,16]. Preserving the designed shape of the DONs and thereby the displayed molecular arrangements under relevant environmental conditions is thus an essential issue for many applications [17].

An important parameter in this regard is the concentration of divalent cations and particularly Mg^2+^. During DON assembly, millimolar concentrations of Mg^2+^ are required in order to screen the electrostatic repulsion of the negatively charged DNA strands and thereby facilitate their compaction into the small volume of the DON. However, such comparatively high Mg^2+^ concentrations are incompatible with numerous applications. For instance, Mg^2+^ ions have been shown to affect drug loading [18,19], mineralization [20,21], and conformational switching and actuation [22,23] of DONs. Fortunately, the synthesized DONs can be transferred post-assembly into appropriate Mg^2+^-free solutions such as phosphate-buffered saline (PBS), Tris buffer, or pure water simply by spin filtering [24]. DON stability in such solutions is maintained by residual Mg^2+^ ions from the folding buffer that remain bound to the DNA’s backbone phosphates upon buffer exchange. This enables the application of DONs in solutions with Mg^2+^ concentrations down to the low-µM range [24].

**Figure 1:**
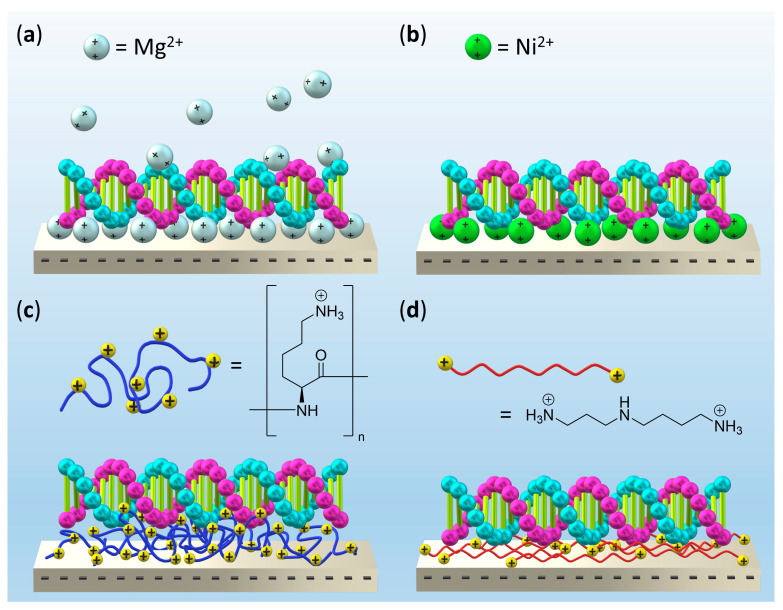
Schematic representations (not to scale) of the different DON adsorption strategies evaluated in this work. (**a**) Addition of Mg^2+^ ions to the DON sample. (**b**) Pre-adsorption of Ni^2+^ ions. (**c**) Pre-adsorption of poly-l-lysine (PLL). (**d**) Pre-adsorption of spermidine (Spdn). The backbone of double-stranded (ds) DNA is represented by cyan and magenta spheres.

However, Mg^2+^ ions serve also another purpose; they facilitate the adsorption of the highly negatively charged DONs at like-charged surfaces such as mica and silicon oxide (see Figure 1a). This is of particular importance in atomic force microscopy (AFM) investigations [5,7], which require ultra-flat substrate surfaces. In such settings, an insufficient Mg^2+^ concentration will result in either high mobility of the adsorbed DONs, which makes them susceptible to post-adsorption manipulation [25,26], or the retardation [27] or even complete suppression [28] of DON adsorption. To avoid these issues, previous studies have mixed the Mg^2+^-free samples with Mg^2+^-containing solutions right before immobilization on mica [24,29,30]. While this approach has proven useful for evaluating the structural integrity of the DONs after exposure to denaturing environments [24,29] or ionizing radiation [30], it is incompatible with the in situ investigation of biomolecular processes such as protein binding [31,32], enzymatic reactions [33,34], or conformational dynamics [35,36] under essentially Mg^2+^-free conditions. In this work, we thus explore alternative means for immobilizing DONs at mica surfaces that do not rely on the addition of Mg^2+^ ions to the DON-containing solution (see Figure 1b–d). For this, 2D DNA origami triangles (DOTs) are synthesized in Mg^2+^-containing TAE buffer and subsequently transferred into either PBS or H_2_O [24]. PBS is as a well-established buffer mimicking physiological pH and ionic strength, while H_2_O was chosen as an ion-free solution, which does not screen any electrostatic interactions. The DOTs are then immobilized from these solutions at mica surfaces with pre-adsorbed Ni^2+^ ions (Figure 1b), poly-l-lysine (PLL, Figure 1c), and spermidine (Spdn, Figure 1d), respectively. The efficiency of DOT adsorption under these conditions and possible effects on the structural integrity of the DOTs are quantified by AFM in the dry state after sample washing and compared to the standard method of Mg^2+^-mediated adsorption (Figure 1a). In PBS, we find that DOT adsorption at PLL- and in particular Spdn-modified mica is superior to Ni^2+^- and even Mg^2+^-mediated adsorption in terms of surface coverage. In pure H_2_O, on the other hand, the situation is markedly different with all three pre-adsorption-based strategies proving inferior to Mg^2+^ addition. Here, PLL performs slightly better than Spdn, with the latter resulting in severe AFM imaging artefacts. For both Mg^2+^-free solutions, however, the Ni^2+^-modified mica surface shows the worst performance, resulting in heavily deformed DOTs. Interestingly, the behavior of tube-like six-helix bundles (6HBs) differed in some regards from that of the DOTs and showed less dramatic differences between the evaluated techniques. This is probably related to the smaller contact area between DON and surface. Our results thus clearly demonstrate the great potential of polyelectrolyte-coated mica to serve as a substrate in DON-based AFM studies under essentially Mg^2+^-free conditions.

## 2. Results and Discussion

### 2.1. Mg^2+^-Mediated Adsorption

**Figure 2:**
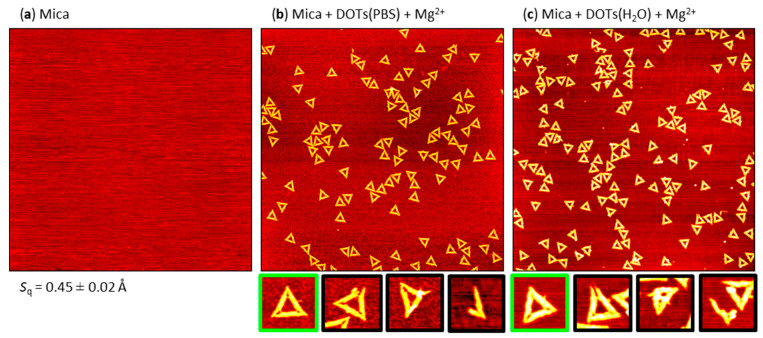
Representative AFM images of (**a**) a freshly cleaved mica surface and DOTs adsorbed from (**b**) PBS and (**c**) H_2_O at the mica surface after addition of 10 mM MgCl_2_ (in 1 × TAE). The AFM images have a size of 3 × 3 µm^2^ and a height scale of (**a**) 1.5 and (**b**,**c**) 2.5 nm, respectively. The RMS roughness *S*q of the freshly cleaved mica surface (average of three AFM images ± standard deviation) is given below the AFM image in (**a**). Below (**b**) and (**c**), example magnifications of intact (green) and damaged (black) DOTs are shown.

In order to establish a benchmark, we first evaluated the efficiency of Mg^2+^-mediated DOT adsorption at freshly cleaved mica surfaces. A representative AFM image of a freshly cleaved mica surface is shown in Figure 2a. The root-mean-square (RMS) surface roughness *S*_q_ of the freshly cleaved mica surface was only about 0.45 Å. Note that this very low value is mostly determined by scanning noise. Adsorption of the DOTs in PBS (pH 7.4) and H_2_O (pH 7.0) at this surface was enabled by mixing the Mg^2+^-free sample solutions immediately before immobilization with folding buffer, i.e., 10 mM MgCl_2_ in 1 × TAE. For both solutions, this resulted in similarly sizeable DOT adsorption (see Figure 2b,c) with an average surface coverage of about 15 adsorbed DOTs per µm^2^.

To identify possible effects of the surface pretreatment on the structural integrity of the DOTs, we also quantified the fractions of intact and damaged DOTs based on an established classification scheme [30,37,38]. In this scheme, the classification “damaged” applies to all DOTs whose shape clearly deviates from the original DOT design (see Ref. [37] for details). This in particular includes DOTs coming apart at the vertices but also broken trapezoids, DOT fragments, severely deformed DOTs, and partially and completely denatured DOTs. The dissociation of the trapezoids composing the Rothemund DOT [1] at the vertices results from the low melting temperatures of the bridging staples and is thus frequently observed in denaturing environments [38,39]. It also represents the predominant type of damage under the current conditions. In contrast to previous classifications, however, we here deliberately excluded slight deformations of the adsorbed DOTs from the category “damaged”. While slight shape deformations represent another very common type of DOT damage under non-denaturing conditions [37,40], they may also be caused by different surface topographies that result from the different pretreatments (see below). For Mg^2+^-mediated adsorption, about 90% of the adsorbed DOTs remained structurally intact, in both PBS and H_2_O. This value is comparable to that observed previously under equivalent conditions [24] as well as in standard folding buffer [37,40].

### 2.2. Pre-Adsorption of Ni^2+^

**Figure 3:**
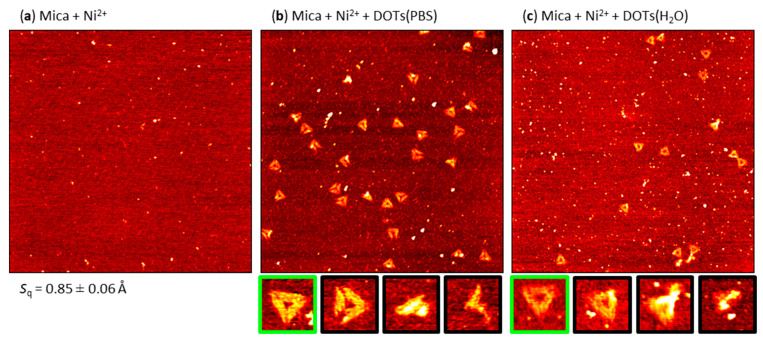
Representative AFM images of (**a**) a mica surface after incubation with NiCl_2_ and DOTs adsorbed from (**b**) PBS and (**c**) H_2_O at the Ni^2+^-modified mica surface. The AFM images have a size of 3 × 3 µm^2^ and a height scale of (**a**) 1.5 and (**b**,**c**) 2.5 nm, respectively. The RMS roughness *S*q of the Ni^2+^-modified mica surface (average of three AFM images ± standard deviation) is given below the AFM image in (**a**). Below (**b**) and (**c**), example magnifications of intact (green) and damaged (black) DOTs are shown.

Next, we evaluated DOT adsorption in the absence of additional Mg^2+^ ions at Ni^2+^-modified mica surfaces. Ni^2+^ ions undergo stronger binding to the mica surface than Mg^2+^. Therefore, mica surfaces pretreated with Ni^2+^ have previously been used for immobilizing genomic DNA molecules [41,42,43,44], while the displacement of Mg^2+^ ions at the mica–DNA interface by Ni^2+^ ions has been employed for the fixation of self-assembled DON lattices [45,46]. Figure 3a shows an AFM image of a mica surface after incubation in 10 mM NiCl_2_ solution. Compared to the freshly cleaved mica surface (see Figure 2a), a much rougher surface with many particle-like features was obtained, possibly as a result of the crystallization of residual NiCl_2_. Consequently, the surface roughness was increased by almost a factor of two to *S*_q_ ~ 0.85 Å.

**Figure 4:**
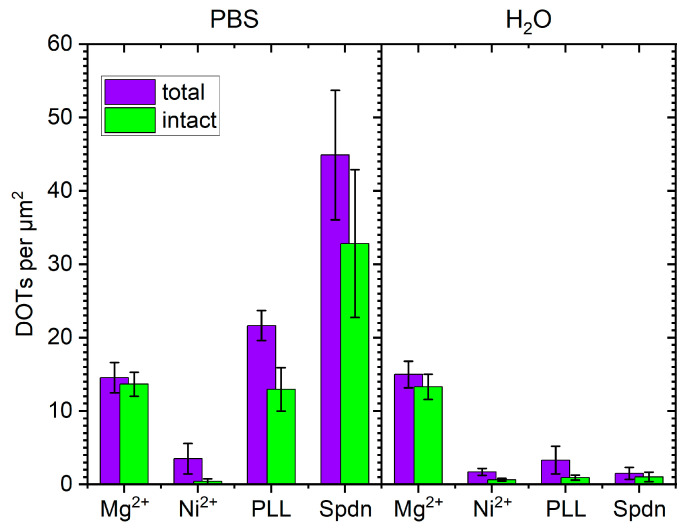
Surface coverage of adsorbed DOTs obtained for the different surface pretreatments and solution conditions. Values represent averages of five to twelve AFM images with the standard deviations as error bars.

As can be seen in Figure 3b,c, DOT adsorption at the Ni^2+^-modified mica surface was strongly reduced compared to Mg^2+^-mediated adsorption. In PBS, the DOT surface coverage was reduced from about 15 DOTs per µm^2^ for Mg^2+^-mediated adsorption to only about 4 DOTs per µm^2^ (see Figure 4). An even lower surface coverage of less than 2 DOTs per µm^2^ was obtained in H_2_O. Furthermore, also the fraction of intact DOTs was drastically decreased at the Ni^2+^-modified mica surface. While about 90% of adsorbed DOTs were intact in the presence of Mg^2+^, adsorption from PBS at the Ni^2+^-modified mica surface yielded only about 12% of intact DOTs (see Table 1). In H_2_O, a higher fraction of intact DOTs of about 37% was obtained. Furthermore, because of the comparatively large roughness of the Ni^2+^-modified mica surface, the intact DOTs had a rather blurred appearance and irregular height (see the magnifications in Figure 3b,c). This will not only hamper their detailed structural characterization but also render the AFM identification of any DON-bound proteins close to impossible.

At first sight, the observation that Ni^2+^ pre-adsorption is not an efficient DON adsorption strategy under Mg^2+^-free conditions may appear surprising because Ni^2+^ is known to have a stronger binding affinity to both DNA [47] and mica [43] than does Mg^2+^. However, Piétrement et al. have shown already in 2003 that efficient immobilization of genomic dsDNA at Ni^2+^-treated mica surfaces requires the presence of Mg^2+^ ions in the sample solution at concentrations of 10 mM or more [42]. At lower Mg^2+^ concentrations of only 2 mM, the authors observed significant mobility of the adsorbed dsDNA molecules. In contrast to the experiments of Piétrement et al., we imaged the adsorbed DOTs in the dry state, which required the washing of the mica surface with ultrapure water to remove non-adsorbed DOTs as well as residual salt (in the case of PBS). It thus appears likely that a large amount of the weakly adsorbed DOTs desorbed from the Ni^2+^-treated mica surface during this washing step, resulting in the observed strongly reduced surface coverage (see Figure 4). This may also provide an explanation for the large number of damaged DOTs found at these surfaces. Piétrement et al. observed that dsDNA molecules, despite their weak adsorption at the Ni^2+^-treated mica surface, resisted complete desorption in the presence of high Na^+^ concentration but instead dangled from the surface [42]. Assuming that the same is true also for the DOTs remaining at the surface during washing, the currents of water flowing along the surface may induce significant distortions of the partially attached DOTs. The resulting stress buildup may then lead to DOT rupture, fragmentation, and structural collapse, as observed in the AFM images shown in Figure 3b,c and Figure S6. It should be noted at this point that the vast majority of in situ AFM studies that employed Ni^2+^-mediated dsDNA or DON adsorption at mica surfaces used DNA-containing buffer solutions supplemented with divalent cations such as Ni^2+^ and Mg^2+^ [48,49,50,51,52,53].

### 2.3. Pre-Adsorption of Poly-l-Lysine (PLL)

**Figure 5:**
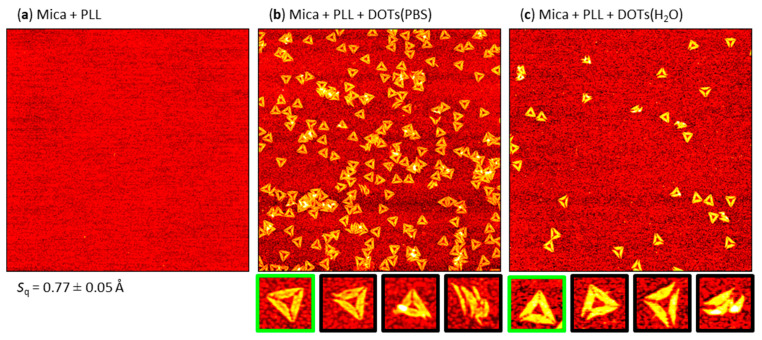
Representative AFM images of (**a**) a mica surface after incubation with PLL and DOTs adsorbed from (**b**) PBS and (**c**) H_2_O at the PLL-modified mica surface. The AFM images have a size of 3 × 3 µm^2^ and a height scale of (**a**) 1.5 and (**b**,**c**) 2.5 nm, respectively. The RMS roughness *S*q of the PLL-modified mica surface (average of three AFM images ± standard deviation) is given below the AFM image in (**a**). Below (**b**) and (**c**), example magnifications of intact (green) and damaged (black) DOTs are shown.

We then turned to polyelectrolyte adsorption for modifying the mica surface. The most prominent polyelectrolyte in the present context is probably PLL, which has been used to immobilize dsDNA molecules [54], DNA-coated nanoparticles [55], and DNA origami [56,57]. The latter, however, was always performed in the presence of millimolar concentrations of Mg^2+^ ions [56,57]. As can be seen in Figure 5a, PLL adsorption at mica resulted in a rather smooth surface topography with an average RMS roughness *S*_q_ below 0.8 Å, which is slightly lower than for the Ni^2+^-treated surface shown in Figure 3a. Furthermore, the surface did not exhibit any particle-like features, in contrast to Ni^2+^-modified mica (cf. Figure 3a).

Exposure of the PLL-modified mica surface to DOTs in PBS resulted in sizeable adsorption (see Figure 5b) with a surface coverage of about 22 DOTs per µm^2^, which is even higher than that obtained for Mg^2+^-mediated adsorption (see Figure 4). The DOTs themselves could be resolved very well and with great structural detail. Closer inspection of the AFM images in Figure 5b and Figure S7a, however, reveals a surprisingly large fraction of damaged DOTs (~40%, see Table 1). In particular, several DOTs can be seen that have disintegrated at their vertices and now consist only of loosely connected trapezoids (see, e.g., Figure 5b, right magnification). This type of damage is only rarely observed for Mg^2+^-mediated adsorption (see Figure 2b and Figure S5a), which indicates either that this damage occurs during adsorption or that such damaged DOTs have a higher affinity for the PLL-modified than for the Mg^2+^-modified surface, so that only the former surface results in their efficient immobilization. Furthermore, we observed a strong tendency of DOT clustering at the PLL-modified surface. Since such clusters are mostly absent at the Mg^2+^-modified mica surface (cf. Figure 2b and Figure S5a), we attribute their formation to the entangled polyelectrolyte layer that probably loops and dangles from the surface into solution (see Figure 1c). Such dangling polyelectrolyte chains may screen the electrostatic repulsion between adsorbed DOTs and thereby facilitate cluster formation.

For DOT adsorption from H_2_O, the situation at the PLL-modified surface was markedly different. As can be seen in Figure 5c, the DOT surface coverage was drastically reduced compared to adsorption from PBS and comparable in magnitude to the surface coverage obtained at Ni^2+^-modified mica (see Figure 4). The appearance of the adsorbed DOTs is very similar to those adsorbed from PBS. However, the fraction of intact DOTs was decreased considerably from about 68% in PBS to only about 28% in H_2_O (see Table 1). This indeed suggests that damaged and intact DOTs have different surface affinities, whose relative magnitude depends on not only the type of surface but also the composition of the surrounding medium. This might again be related to the soft, cushion-like nature of the entangled PLL layer (see Figure 1c), which may partially enclose the adsorbed DOTs and thereby better accommodate the more three-dimensional shapes of damaged DOTs. This would not only result in better adhesion but also protect the adsorbed DOTs during sample washing.

### 2.4. Pre-Adsorption of Spermidine (Spdn)

**Figure 6:**
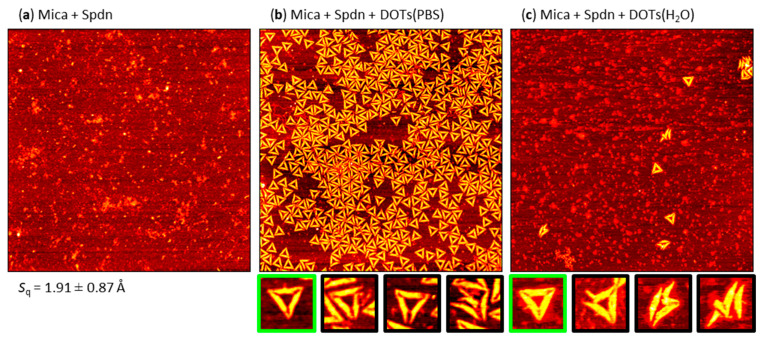
Representative AFM images of (**a**) a mica surface after incubation with Spdn and DOTs adsorbed from (**b**) PBS and (**c**) H_2_O at the Spdn-modified mica surface. The AFM images have a size of 3 × 3 µm^2^ and a height scale of (**a**) 1.5 and (**b**,**c**) 2.5 nm, respectively. The RMS roughness *S*q of the Spdn-modified mica surface (average of three AFM images ± standard deviation) is given below the AFM image in (**a**). Below (**b**) and (**c**), example magnifications of intact (green) and damaged (black) DOTs are shown.

Because of the observed clustering of the adsorbed DOTs and the larger fraction of damaged DOTs at the PLL-modified mica surface, we next tested Spdn as a shorter polyelectrolyte that can also be used to adsorb DNA [58] but should not form such an entangled polyelectrolyte layer (see Figure 1d). However, as can be seen in Figure 6a, the resulting surface had a very pronounced topography dominated by small particles and large islands, which suggests the buildup of multilayers. Consequently, the Spdn-modified mica surface by far had the largest RMS roughness of all the surfaces studied in this work, i.e., *S*_q_ ~ 1.9 Å. Despite this rough surface topography, surprisingly strong DOT adsorption from PBS solution can bes observed in the AFM images in Figure 6b and Figure S8a. At a value of 45 DOTs per µm^2^, the achieved DOT surface coverage was about thrice as high as for Mg^2+^-mediated adsorption (see Figure 4). While the fraction of intact DOTs of about 73% was smaller than for Mg^2+^-mediated adsorption (see Table 1), the appearance of the DOTs was comparable. Spdn pre-adsorption is thus vastly superior to the other Mg^2+^-free methods evaluated in this study for immobilizing DOTs from PBS. For DOTs suspended in H_2_O, however, a similar behavior was observed as for PLL-modified mica, i.e., vastly reduced adsorption with a surface coverage of only about 1.5 DOTs per µm^2^ (see Figure 6c and Figure S8b). Even though the fraction of intact DOTs was more than twice as high as for PLL, i.e., about 68% (see Table 1), the Spdn-modified surface proved very difficult to image after DOT adsorption from H_2_O, with the recorded AFM images often showing severe imaging artefacts (see Figure S8b). Therefore, immobilizing DOTs from H_2_O at Spdn-modified mica surfaces is not particularly compatible with AFM investigations.

### 2.5. Effect of PBS and H_2_O Exposure on the Pre-Adsorbed Polyelectrolyte Films

The observation that DOT adsorption at both polyelectrolyte-modified surfaces is much weaker in H_2_O than in PBS is rather surprising. Because of its comparatively high ionic strength, PBS has a Debye length of only about 0.7 nm [59], so that any long-range electrostatic interactions between the negatively charged DOTs and the positively charged polyelectrolyte films will be efficiently screened. In the absence of any ions, one would thus expect a stronger interaction. Obviously, this was not the case in the present experiments. On the other hand, the same ions will also screen the electrostatic repulsion between neighboring (protonated) amino groups in the polyelectrolyte layer. Exposure to PBS may thus lead to structural reorganizations inside the adsorbed polyelectrolyte film and thereby a more compact and homogeneous surface layer. Furthermore, PBS contains a comparatively large concentration of Na^+^ ions, which can specifically interact with the exposed mica surface and thereby neutralize its negative charge [60,61], resulting in reduced electrostatic repulsion between DOTs and exposed areas of the mica surface showing through. In H_2_O, this repulsion will remain unscreened.

**Figure 7:**
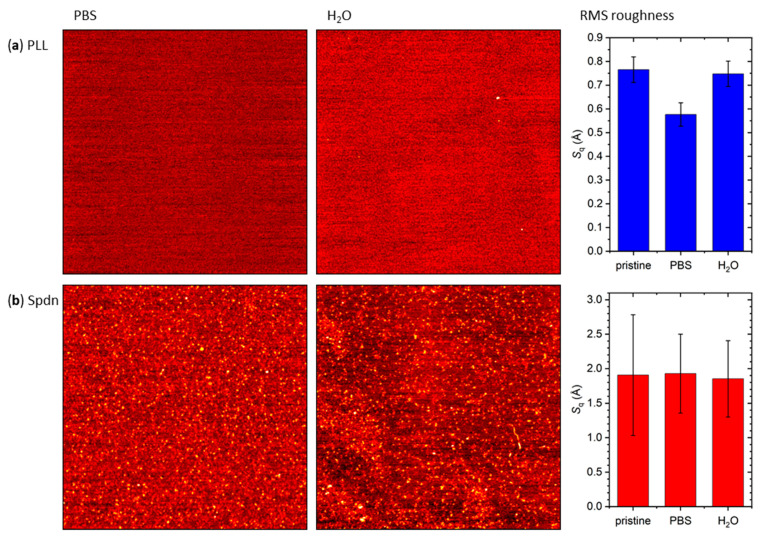
Representative AFM images of (**a**) PLL- and (**b**) Spdn-modified mica surfaces after incubation in PBS (left) and H_2_O (center). The right panels provide the RMS surface roughness values before and after incubation. The AFM images have a size of 3 × 3 µm^2^ and a height scale of 1.5 nm. The RMS roughness values represent averages of three AFM images with the standard deviations as error bars.

In order to obtain more insights into this effect, we exposed both polyelectrolyte-modified surfaces to PBS and H_2_O without DOTs and evaluated the effect on the surface topography. As can be seen in Figure 7a, exposure of the PLL-modified mica surface to PBS indeed resulted in a reduction of the RMS surface roughness, even though no morphological differences were visible in the corresponding AFM images. This reduction in *S*_q_ is indicative of a compaction of the adsorbed PLL film. Exposure to H_2_O, on the other hand, did not have any detectable effect on the morphology of the PLL film. This was to be expected since the mica pretreatment utilized PLL dissolved in H_2_O (see Section 3.2).

In the case of Spdn, no effect of exposure to PBS or H_2_O on the RMS surface roughness was observed (see Figure 7b). This can be attributed to the fact that the Spdn surface was very rough and inhomogeneous to begin with (see Figure 6a and Figure S4a). Nevertheless, visual inspection of the AFM images in Figure 6a, Figure 7b and Figure S4 suggests some minor morphological transition upon exposure to PBS. In particular, the Spdn film appeared more homogeneous over micrometer length scales after exposure to PBS and did not show such a pronounced island topography anymore. In sum, these observations indeed suggest that efficient DNA origami immobilization at polyelectrolyte-modified mica surfaces requires a certain ionic strength in order to create a compact and homogeneous polyelectrolyte film.

### 2.6. Effect of DON Shape

**Figure 8:**
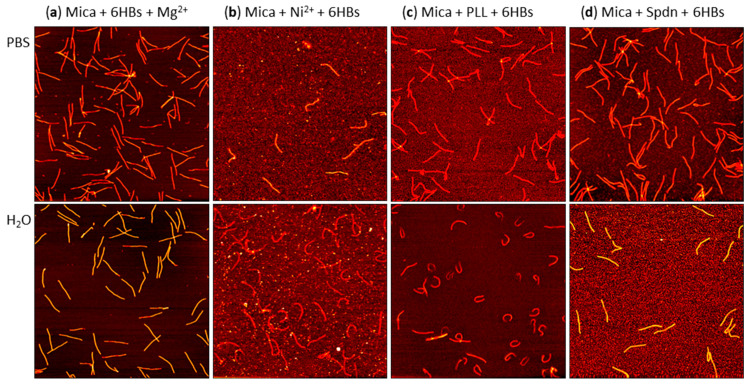
Representative AFM images of 6HB DONs adsorbed at mica surfaces from PBS (upper row) and H_2_O (lower row) via (**a**) Mg^2+^ addition, (**b**) Ni^2+^ pretreatment, (**c**) PLL pretreatment, and (**d**) Spdn pretreatment. The AFM images have a size of 3 × 3 µm^2^ and a height scale of 4 nm.

**Figure 9:**
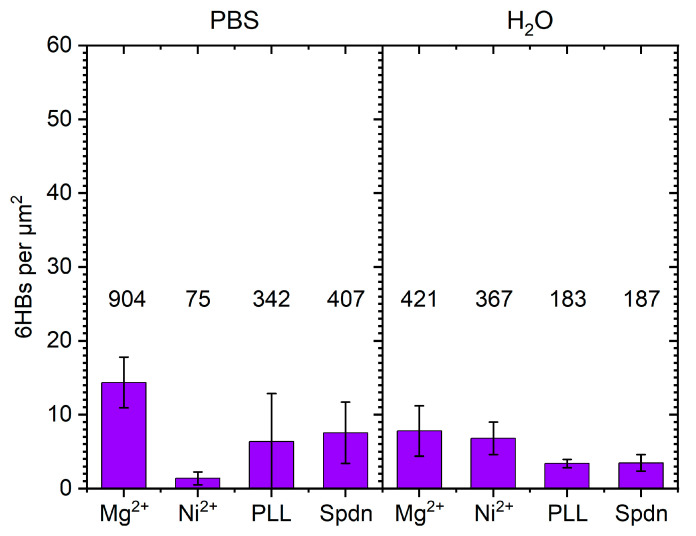
Surface coverage of adsorbed 6HBs obtained for the different surface pretreatments and solution conditions. Values represent averages of six AFM images with the standard deviations as error bars. The total number of counted 6HBs used in the statistical analysis is given above each bar.

In order to assess the generality of the above observations, we repeated these experiments with a rather different DON shape, i.e., a tube-like 6HB [62], even though this shape has only very limited relevance as a substrate for single-molecule AFM investigations. As can be seen in Figure 8, much smaller differences in surface coverage were observed compared to the 2D DOTs. Since the shape of the 6HBs is less distinct than that of the DOTs, identification of structural damage is rather challenging. Therefore, we only determined the total number of 6HBs per µm^2^, without any distinction between damaged and intact DONs. The results of the statistical analyses are shown in Figure 9 and support the qualitative observations based on the AFM images in Figure 8.

The observation that the 6HBs did not show such drastic differences in surface coverage between the different immobilization methods as the DOTs most likely resulted from their different surface footprints. Arranging the individual DNA double helices not in a 2D sheet but rather a 3D tube results in a smaller contact area between the 6HBs and the mica surface. Therefore, adsorption of the 6HB will be less sensitive to lateral variations in the pre-adsorbed film. On the other hand, efficient adsorption of the tube-like 6HBs due to electrostatic interactions is usually accompanied by their structural collapse and, thus, flattening at the surface, which results in an increased contact area. Whether this is possible or not will depend on the strength of the DNA–surface interactions and thus on the type of surface modification.

Furthermore, the data in Figure 9 suggest that for the 6HBs, Mg^2+^-mediated adsorption was less efficient in H_2_O than in PBS, whereas the opposite was observed for Ni^2+^ pretreatment. In contrast, no dependence of Mg^2+^- and Ni^2+^-mediated adsorption on the solution conditions was observed for the DOTs (see Figure 4). This may be related to superstructure-specific differences in the interactions between DONs and the different ions (both in solution and at the surface), as previously observed for Mg^2+^ and Eu^3+^ ions coordinating to the backbone phosphates of DOTs and 6HBs [63]. Finally, in H_2_O, the 6HBs adsorbed at the PLL-modified mica surface in a strongly curved conformation (see Figure 8c and Figure S11b). To a lesser degree, this was also observed for the Ni^2+^-modified surface in the same solution (see Figure 8b and Figure S10b). This may be related to variations in the mechanical properties and in particular the flexibility of the 6HBs suspended in the different solutions. In H_2_O, the electrostatic repulsion between neighboring double helices will be more pronounced than in high-ionic strength PBS. This may lead to differences in the relaxation of residual strain and in turn to different global conformations. However, since such curved conformations are not observed at the Spdn-modified surface under the same conditions, the surface properties obviously have a strong influence as well. While elucidating these superstructure-specific effects on surface coverage and conformation of the adsorbed DONs will require further study, we can already conclude that applying polyelectrolyte coatings on mica surfaces is a viable strategy for immobilizing DONs at mica surfaces under essentially Mg^2+^-free conditions.

## 3. Materials and Methods

### 3.1. DON Assembly and Buffer Exchange

DOTs [1] and 6HBs [62] were assembled as previously described [24] by using the 7249-nt long M13mp18 scaffold strand (Tilibit GmbH, München, Germany) and 208 and 170 staple strands (Eurofins Genomics GmbH, Ebersberg, Germany), respectively, in 1 × TAE buffer (Carl Roth GmbH + Co. KG, Karlsruhe, Germany) containing 10 mM MgCl_2_ (Sigma-Aldrich Chemie GmbH, Steinheim, Germany). The mixtures were rapidly heated to 80 °C, followed by slow cooling to room temperature within 90 min in a Thermocycler Primus 25 advanced (PEQLAB Biotechnologie GmbH, Erlangen, Germany). The folding buffer was exchanged during purification by spin filtering (Amicon Ultra, 100K, Merck KGaA, Darmstadt, Germany) with HPLC-grade water (pH 7.0, VWR International S.A.S., Fontenay-sous-Bois, France) and PBS buffer (138 mM sodium chloride, 2.7 mM potassium chloride, 10 mM sodium phosphate, 2.7 mM potassium phosphate, pH 7.4, Sigma-Aldrich Chemie GmbH, Steinheim, Germany), respectively. As was shown previously, this buffer exchange does not induce any additional structural damage to the DONs [24]. The resulting DNA origami concentration was determined using a Nanophotometer P 330 (Implen GmbH, München, Germany).

### 3.2. Mica Surface Modification

Mica substrates (Ted Pella, Inc., Redding, CA, USA) were pretreated with NiCl_2_ (Sigma-Aldrich Chemie GmbH, Steinheim, Germany), PLL hydrobromide (molecular weight 1000–5000, Sigma-Aldrich Chemie GmbH, Steinheim, Germany), and Spdn (Alfa Aesar, Thermo Fisher (Kandel) GmbH, Kandel, Germany), respectively.

(1) NiCl_2_ pretreatment: 10 mM NiCl_2_ aqueous solution was deposited onto a freshly cleaved mica surface and incubated for 1 h. An incubation time of 1 h was chosen based on our previous work [45]. It should be noted, however, that equivalent results as reported here were also obtained with shorter incubation times, i.e., 1 min to 30 min. The mica substrate was then rinsed with HPLC-grade water to remove excess NiCl_2_.
(2) PLL pretreatment: PLL was dissolved in HPLC-grade water to yield a 0.1% *w/v* PLL solution. The PLL solution was deposited onto a freshly cleaved mica surface and incubated for 1 h. An incubation time of 1 h was chosen based on literature to ensure maximum surface coverage [64]. The mica substrate was then rinsed with HPLC-grade water to remove excess PLL.
(3) Spdn pretreatment: Spdn was dissolved in HPLC-grade water to yield a 5 mg/mL Spdn solution and then deposited onto a freshly cleaved mica surface. After incubation for 5 min the mica substrate was rinsed with HPLC-grade water. An incubation time of 5 min was chosen based on literature [58].

### 3.3. DON Immobilization and AFM Imaging

For DON immobilization, 2 nM DONs in HPLC-grade water and PBS were incubated on the pretreated mica surfaces for 1 min, respectively. For the reference experiments shown in Figure 7, the same protocol was used but without DONs. For Mg^2+^-mediated adsorption, the concentrated DON solutions in H_2_O and PBS were diluted to 2 nM with 1 × TAE buffer containing 10 mM MgCl_2_, respectively, and incubated on a freshly cleaved mica surface for 1 min. After incubation, the mica substrates were rinsed with HPLC-grade water and blow-dried in a stream of ultrapure air. AFM measurements were carried out by using an Agilent 5500 and a JPK NanoWizard III AFM in intermittent contact mode in air with silicon cantilevers (MikroMasch HQ:NSC18/Al BS, NanoAndMore GmbH, Wetzlar, Germany). Images were recorded with scan sizes of 3 × 3 μm^2^ and a resolution of 1024 px × 1024 px.

The numbers of intact and damaged DOTs visible in the AFM images were counted manually and averaged over five to twelve AFM images recorded under equivalent conditions. The established AFM-based classification of DOTs into intact, broken, denatured, and deformed was applied [30,37]. However, because of the different RMS roughness values obtained after the different pretreatments, only severely deformed DOTs were considered damaged, while slightly deformed ones were treated as intact. Because structural damage is more difficult to assess for the 6HBs [37], we only counted the total numbers of adsorbed 6HBs without any distinction between intact and damaged DONs. The few 6HB fragments sometimes observed were not considered at all. Each experiment was performed twice, and AFM images from both experiments were included in the statistical analyses.

## 4. Conclusions

In this work, we investigated the immobilization of 2D DOTs and tube-like 6HBs at mica surfaces under essentially Mg^2+^-free conditions using different surface pretreatments. For the DOTs, pre-adsorption of Ni^2+^ ions showed the worst performance in both PBS and H_2_O, with low surface coverage, large fractions of damaged DOTs, and severely distorted intact DOTs. In contrast, polyelectrolyte coatings showed vastly superior performance in PBS. DOT adsorption at the Spdn-modified mica surface in particular resulted in greatly enhanced surface coverage compared to Mg^2+^-mediated adsorption, with about 70% of the adsorbed DONs remaining intact. While PLL-modified mica performed more similarly to Mg^2+^ addition in terms of surface coverage, the adsorbed DOTs could be resolved in much greater detail, rendering this surface particularly promising for structural DON characterization. For DOTs suspended in H_2_O, however, the performance of both polyelectrolyte coatings was greatly reduced, in terms of both surface coverage and fraction of intact DONs. Furthermore, AFM imaging of the DOTs adsorbed at the Spdn-modified surface proved particularly difficult under these conditions and resulted in severe imaging artefacts, which renders PLL-mediated DOT adsorption a better choice for AFM-based investigations in H_2_O.

The counterintuitive behavior of the polyelectrolyte films in the different Mg^2+^-free solutions is attributed to electrostatic repulsion between the adsorbed polyelectrolyte chains at low ionic strength, leading to a partially exposed mica surface, which is in this case not screened by the buffer solution. In PBS, the repulsive interactions between the adsorbed molecules are efficiently screened, which results in more compact and homogeneous polyelectrolyte films along with a screening of the charge of the partially exposed mica. Both effects promote enhanced DOT adsorption.

Finally, we investigated also the role of DON shape and found that the differences between the different strategies observed for the adsorption of 2D DOTs are less pronounced for 3D 6HBs. Here, all Mg^2+^-free strategies produce rather similar surface coverage, while PLL- and Ni^2+^-mediated adsorption both result in strongly curved 6HBs. Despite these superstructure-specific differences, polyelectrolyte-coated mica surfaces represent suitable and versatile substrates for DON immobilization under essentially Mg^2+^-free conditions. Our results thus provide some general guidance for the efficient immobilization of DONs at mica surfaces under Mg^2+^-free conditions and may thus aid future in situ AFM studies of biomolecular reactions and dynamics.

## Figures and Tables

**Table 1:** Absolute numbers of total, intact, and damaged DOTs evaluated for each immobilization strategy with the resulting percentage of intact DOTs.

Strategy	Solution	Total	Intact	Damaged	Percentage Intact
Mg^2+^	PBS	654	614	40	93.9
	H_2_O	808	717	91	88.7
Ni^2+^	PBS	315	38	277	12.1
	H_2_O	92	34	58	37.0
PLL	PBS	1557	932	625	59.9
	H_2_O	179	49	130	27.4
Spdn	PBS	3635	2657	978	73.1
	H_2_O	161	110	51	68.3

## Data Availability

The data presented in this study are available on request from the corresponding author.

## Supplementary Materials

The following are available online. Figure S1: Additional AFM images of the freshly cleaved mica surface, Figure S2: Additional AFM images of the Ni^2+^-modified mica surface, Figure S3: Additional AFM images of the PLL-modified mica surface before (a) and after exposure to PBS (b) and H_2_O (c), Figure S4: Additional AFM images of the Spdn-modified mica surface before (a) and after exposure to PBS (b) and H_2_O (c), Figure S5: Additional AFM images of DOTs adsorbed from PBS (a) and H_2_O (b) via Mg^2+^ addition at the mica surface, Figure S6: Additional AFM images of DOTs adsorbed from PBS (a) and H_2_O (b) at the Ni^2+^-modified mica surface, Figure S7: Additional AFM images of DOTs adsorbed from PBS (a) and H_2_O (b) at the PLL-modified mica surface, Figure S8: Additional AFM images of DOTs adsorbed from PBS (a) and H_2_O (b) at the Spdn-modified mica surface, Figure S9: Additional AFM images of 6HBs adsorbed from PBS (a) and H_2_O (b) via Mg^2+^ addition at the mica surface. Figure S10: Additional AFM images of 6HBs adsorbed from PBS (a) and H_2_O (b) at the Ni^2+^-modified mica surface, Figure S11: Additional AFM images of 6HBs adsorbed from PBS (a) and H_2_O (b) at the PLL-modified mica surface, Figure S12: Additional AFM images of 6HBs adsorbed from PBS (a) and H_2_O (b) at the Spdn-modified mica surface.

## Sample Availability

Samples of the compounds are not available from the authors.
