# Supplementary material for: Magnesium-Free Immobilization of DNA Origami Nanostructures at Mica Surfaces for Atomic Force Microscopy
Source: Molecules. 2021 Aug 7;26(16):4798. doi: 10.3390/molecules26164798 (PMC8399889; doi:10.3390/molecules26164798)
Supplement: Supplementary file 1 [file molecules-26-04798-s001.zip › molecules-1318198-supplementary.pdf]

Supplementary Materials

Magnesium-Free Immobilization of DNA Origami Nanostructures at Mica Surfaces for Atomic Force Microscopy

Yang Xin, Amir Ardalan Zargariantabrizi, Guido Grundmeier and Adrian Keller *

Technical and Macromolecular Chemistry, Paderborn University, Warburger Str. 100, 33098 Paderborn, Germany;
yangxin@mail.uni-paderborn.de (Y.X.); ardalan@mail.uni-paderborn.de (A.A.Z.); g.grundmeier@tc.uni-paderborn.de (G.G.)
* Correspondence: adrian.keller@uni-paderborn.de

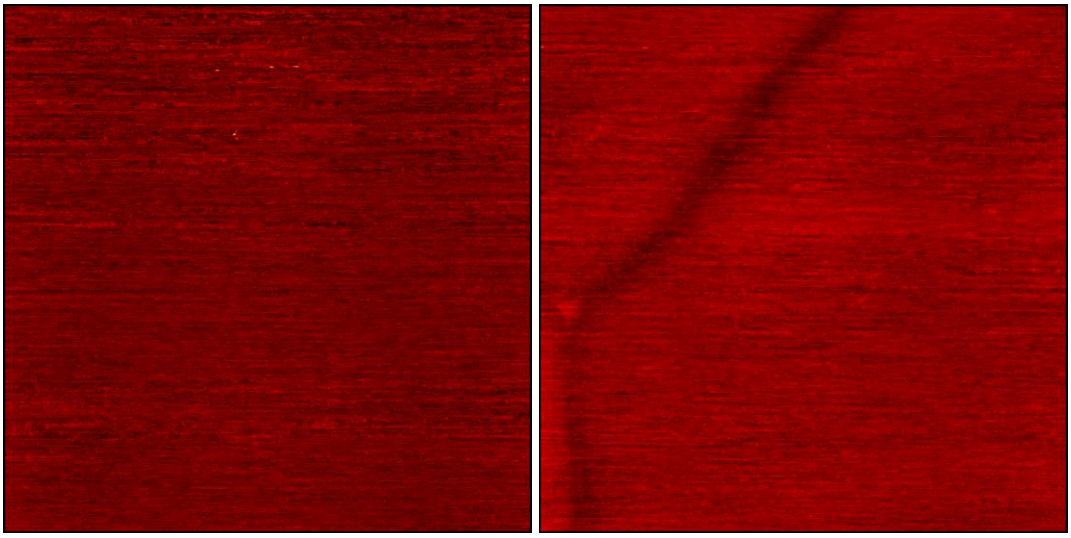

Figure S1: *Additional AFM images of the freshly cleaved mica surface. Images are* $3 \times 3\ \mu m^2$ *and have a height scale of 1.5 nm.*

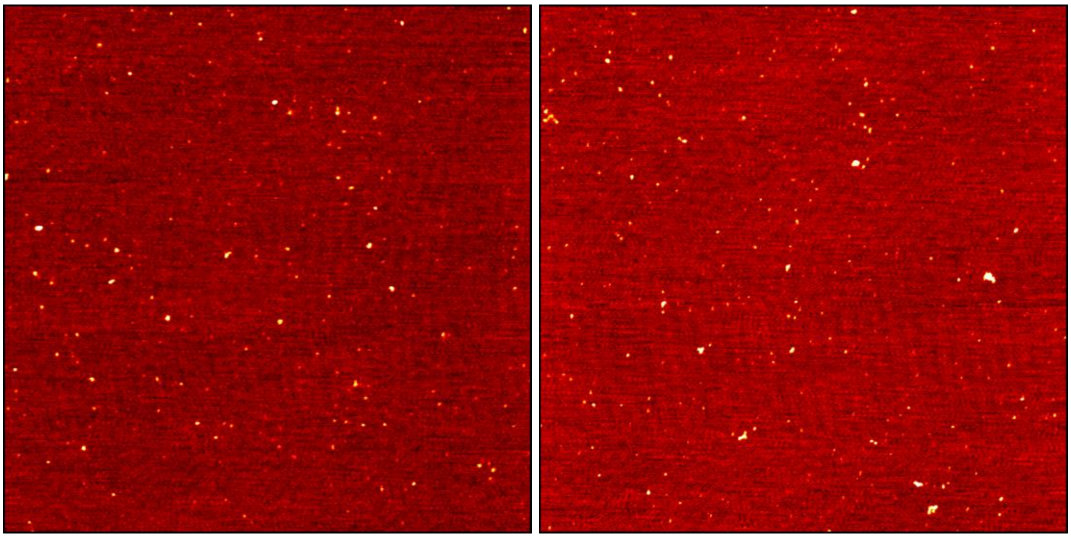

Figure S2: *Additional AFM images of the* Ni^{2+}*-modified mica surface. Images are* $3 \times 3\ \mu m^2$ *and have a height scale of 1.5 nm.*

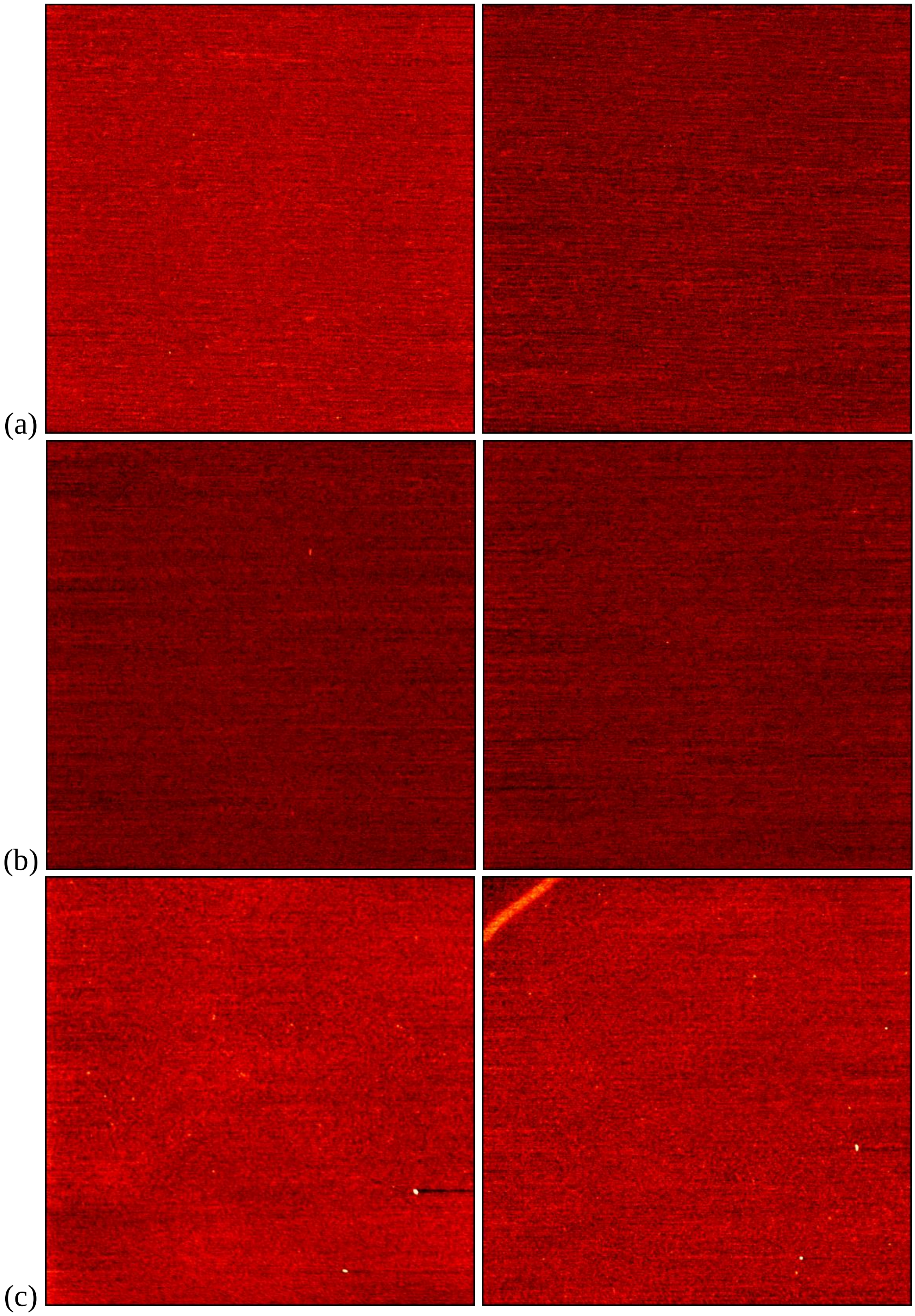

Figure S3: *Additional AFM images of the PLL-modified mica surface before (a) and after exposure to PBS (b) and H_2O (c). Images are $3 \times 3\ \mu m^2$ and have a height scale of 1.5 nm.*

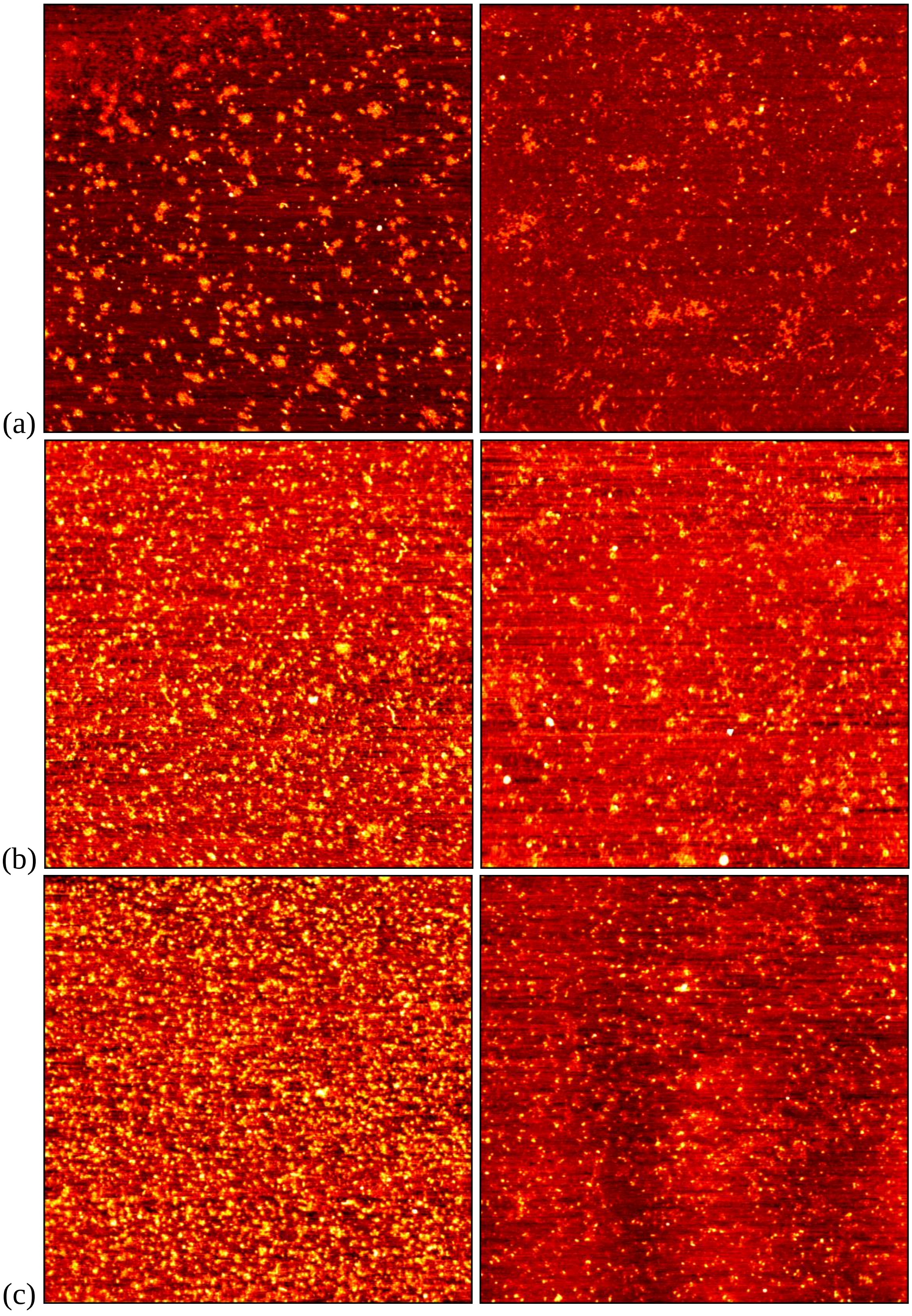

Figure S4: *Additional AFM images of the Spdn-modified mica surface before (a) and after exposure to PBS (b) and H_2O (c). Images are 3 × 3 μm ²and have a height scale of 1.5 nm.*

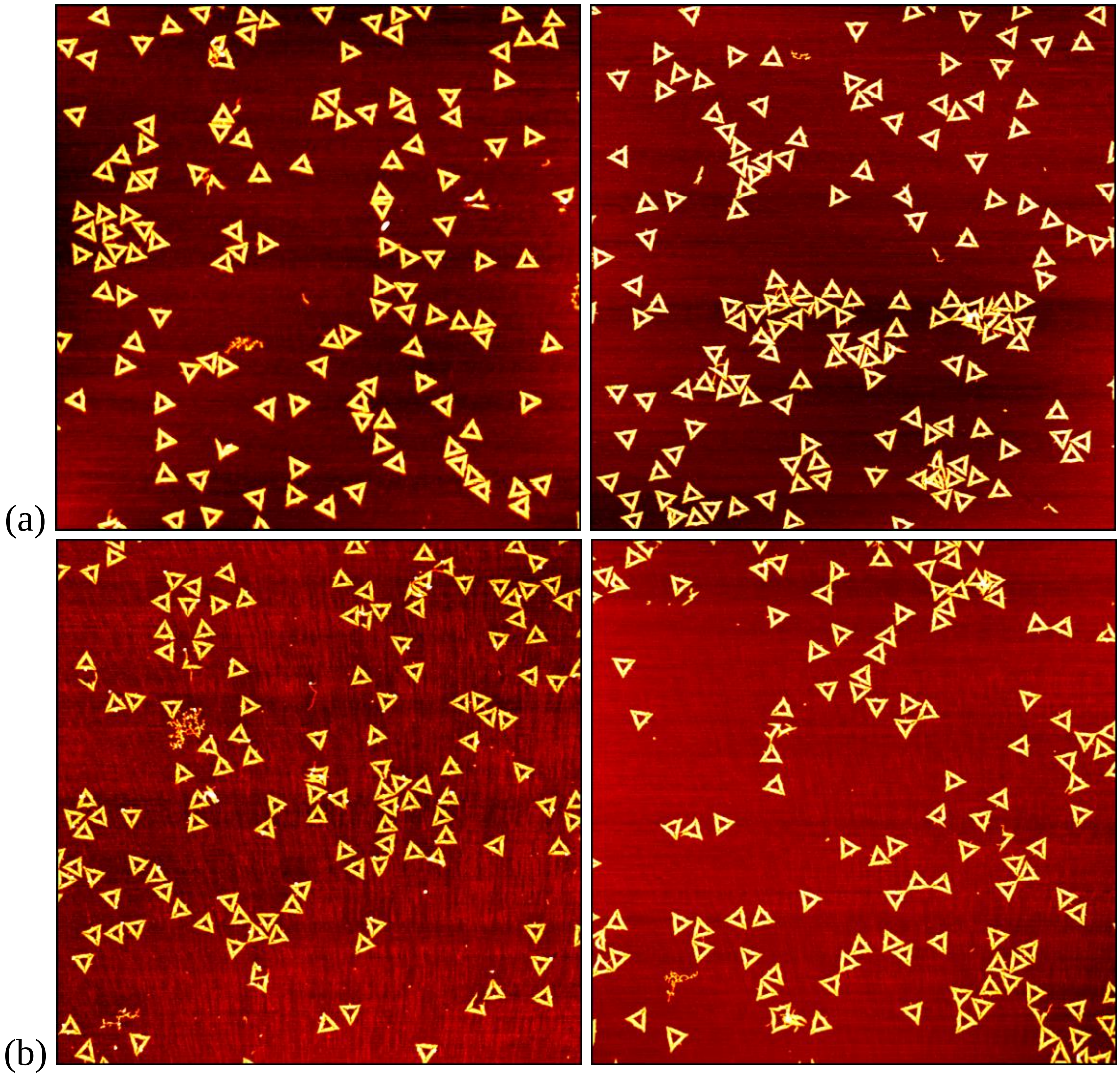

Figure S5: *Additional AFM images of DOTs adsorbed from PBS (a) and* H_2O *(b) via* Mg^{2+} *addition at the mica surface. Images are 3 × 3 μm*^{2} *and have a height scale of 2.5 nm.*

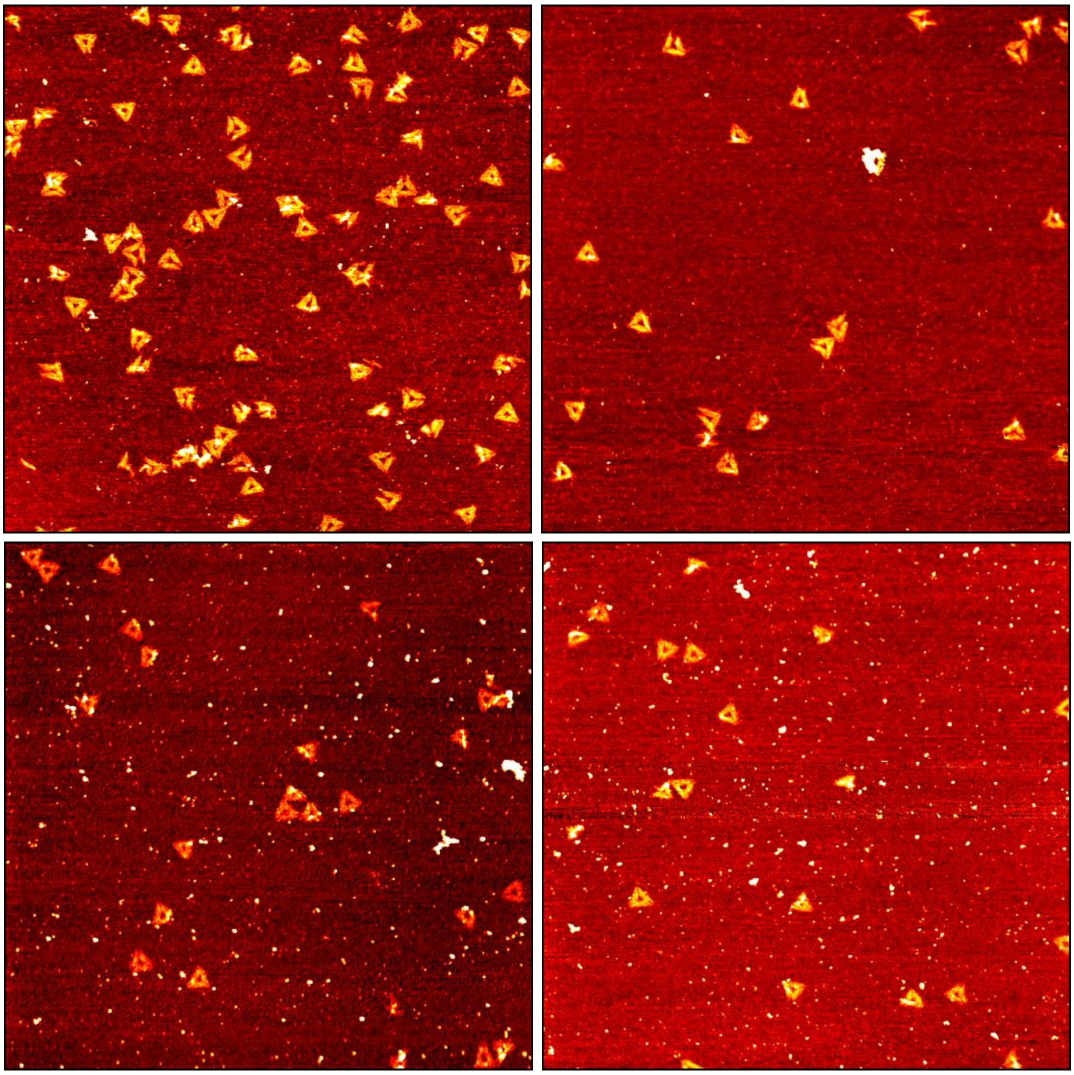

(a)

(b)

Figure S6: *Additional AFM images of DOTs adsorbed from PBS (a) and* H_2O *(b) at the* Ni^{2+}*-modified mica surface. Images are 3 × 3* μm^2 *and have a height scale of 2.5 nm.*

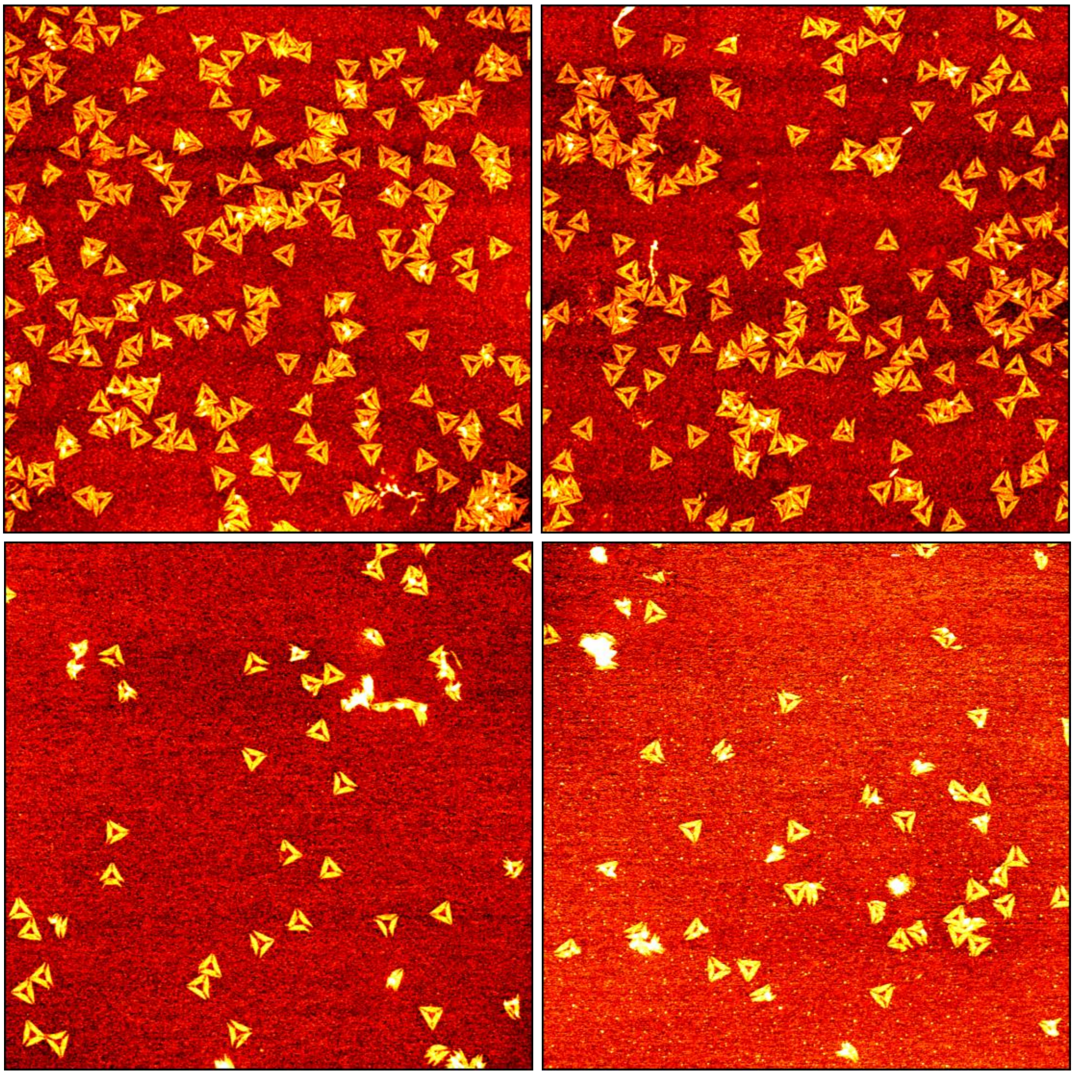

(a)

(b)

Figure S7: *Additional AFM images of DOTs adsorbed from PBS (a) and H_2O (b) at the PLL-modified mica surface. Images are 3 × 3 μm* ^{2}*and have a height scale of 2.5 nm.*

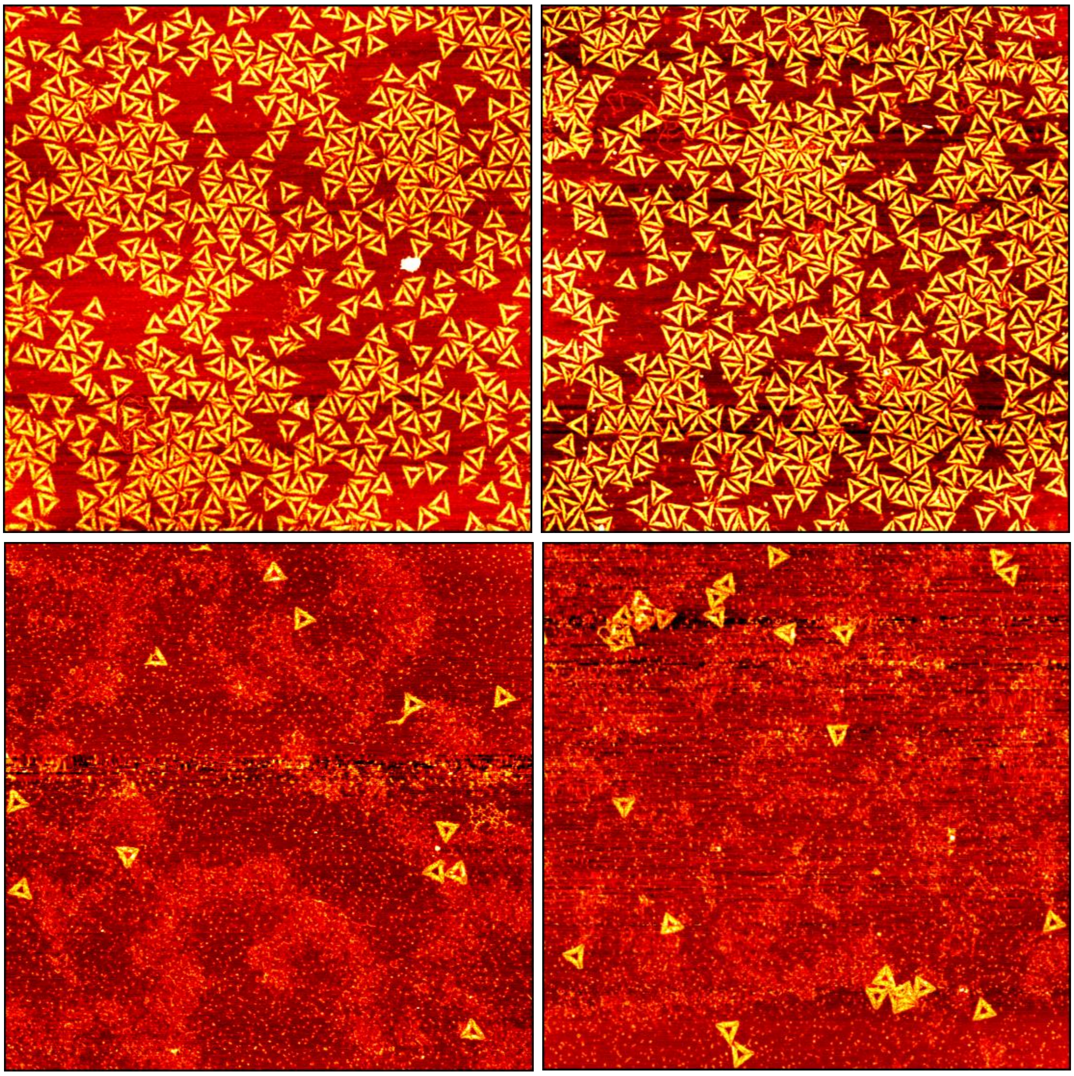

(a)

(b)

Figure S8: *Additional AFM images of DOTs adsorbed from PBS (a) and H_2O (b) at the Spdn-modified mica surface. Images are 3 × 3 μm ²and have a height scale of 2.5 nm.*

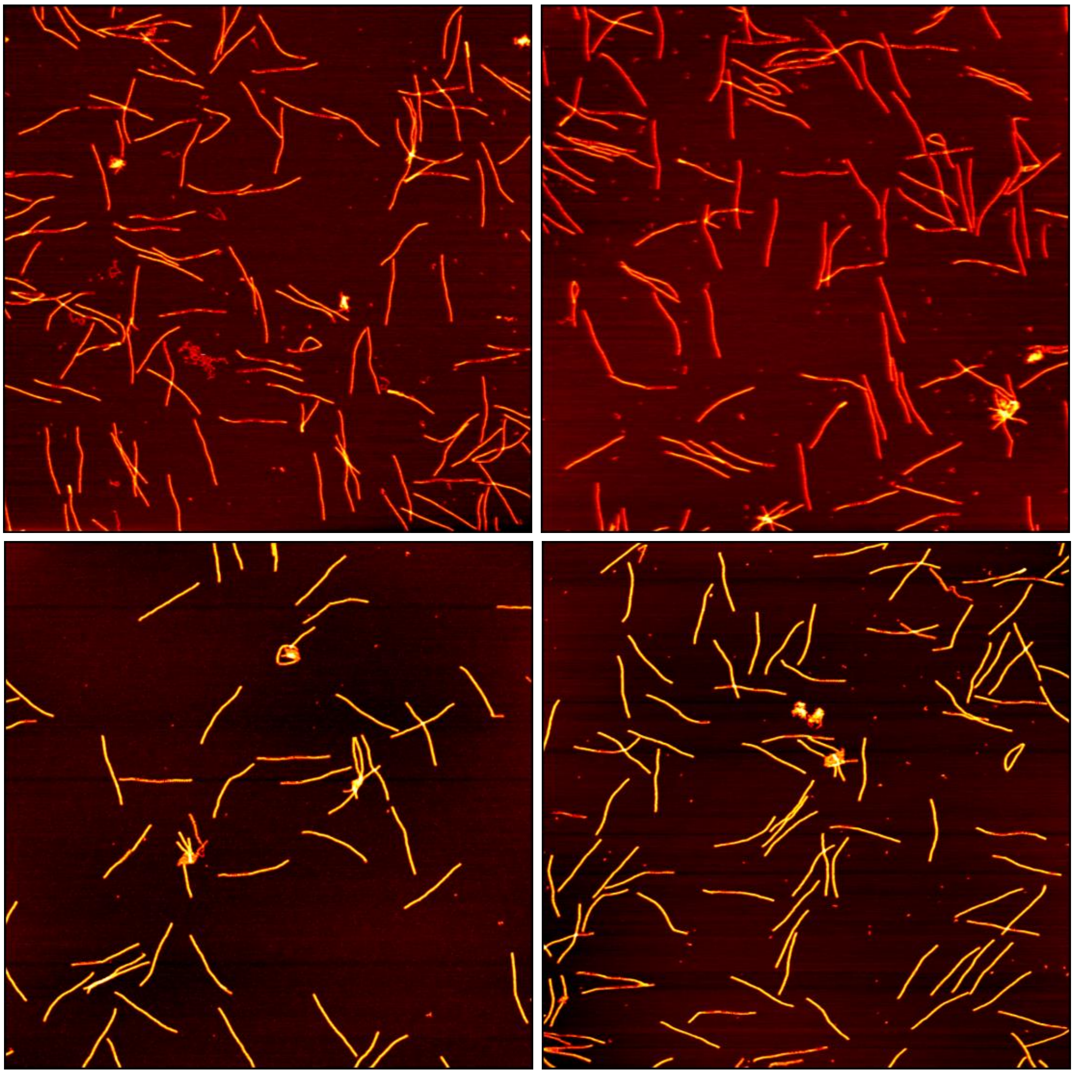

(a)

(b)

Figure S9: *Additional AFM images of 6HBs adsorbed from PBS (a) and H_2O (b) via Mg^{2+} addition at the mica surface. Images are 3 × 3 μm ²and have a height scale of 4 nm.*

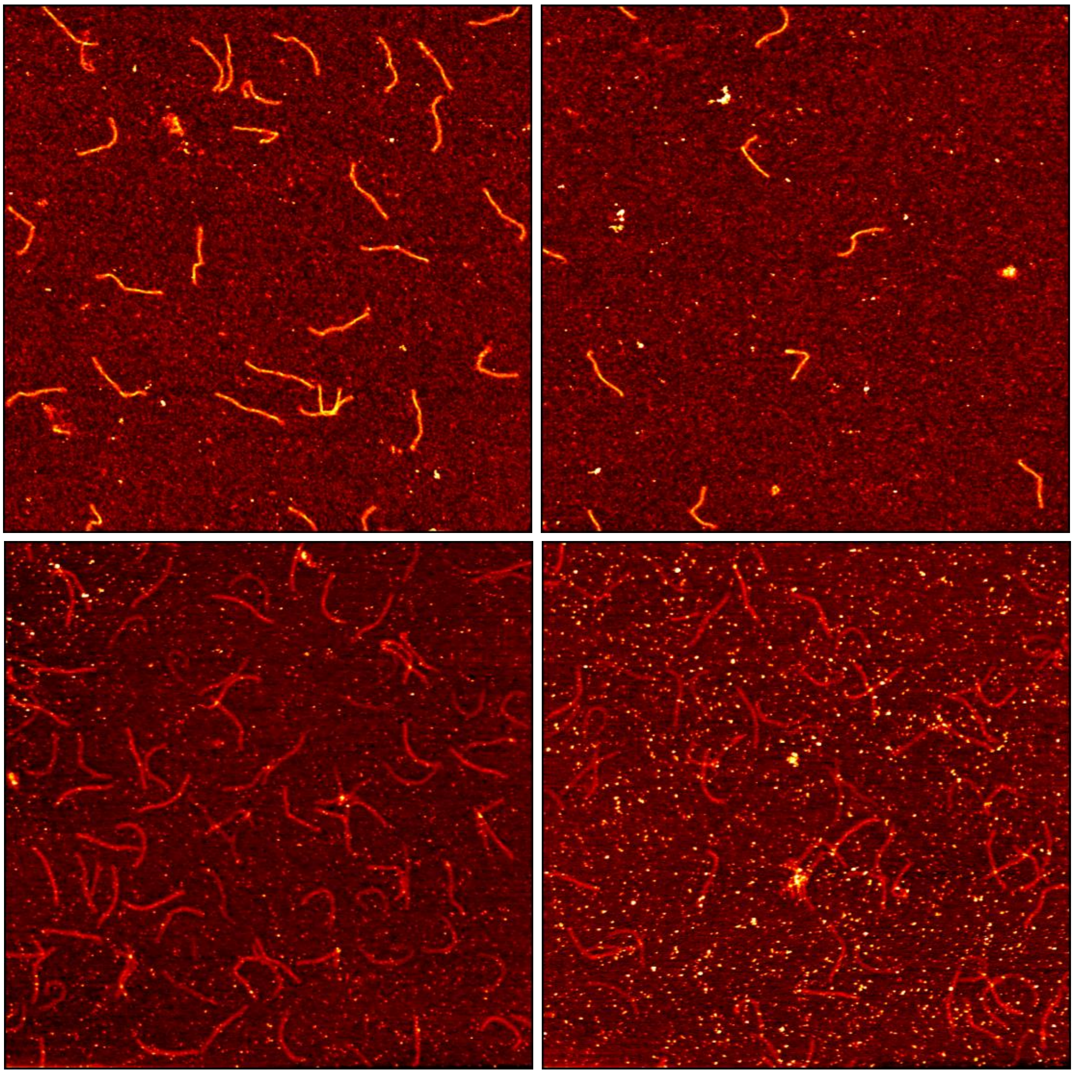

(a)

(b)

Figure S10: *Additional AFM images of 6HBs adsorbed from PBS (a) and H_2O (b) at the Ni^{2+}-modified mica surface. Images are 3 × 3 µm ²and have a height scale of 4 nm.*

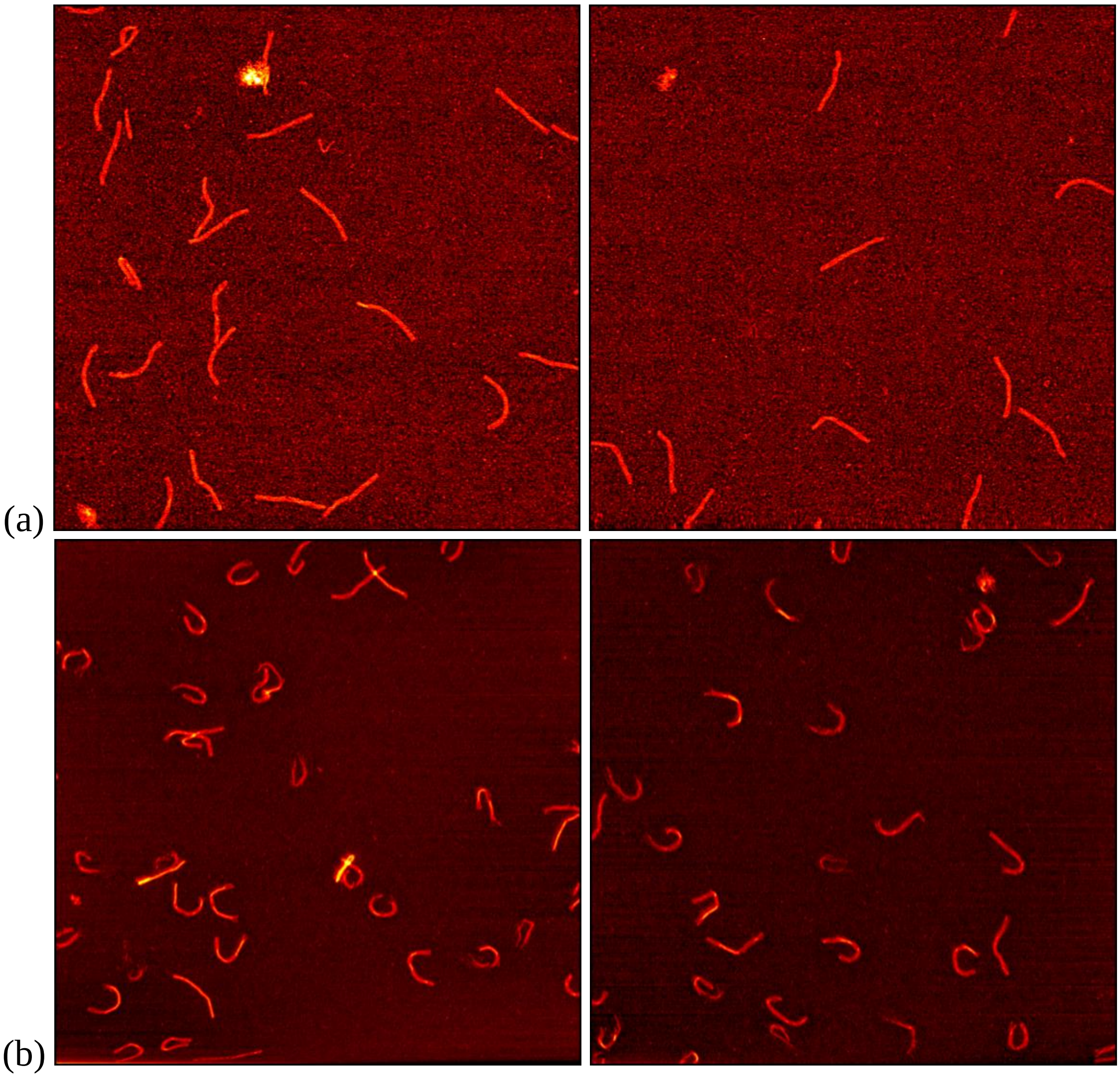

Figure S11: *Additional AFM images of 6HBs adsorbed from PBS (a) and H_2O (b) at the PLL-modified mica surface. Images are 3 × 3 µm ^{2} and have a height scale of 4 nm.*

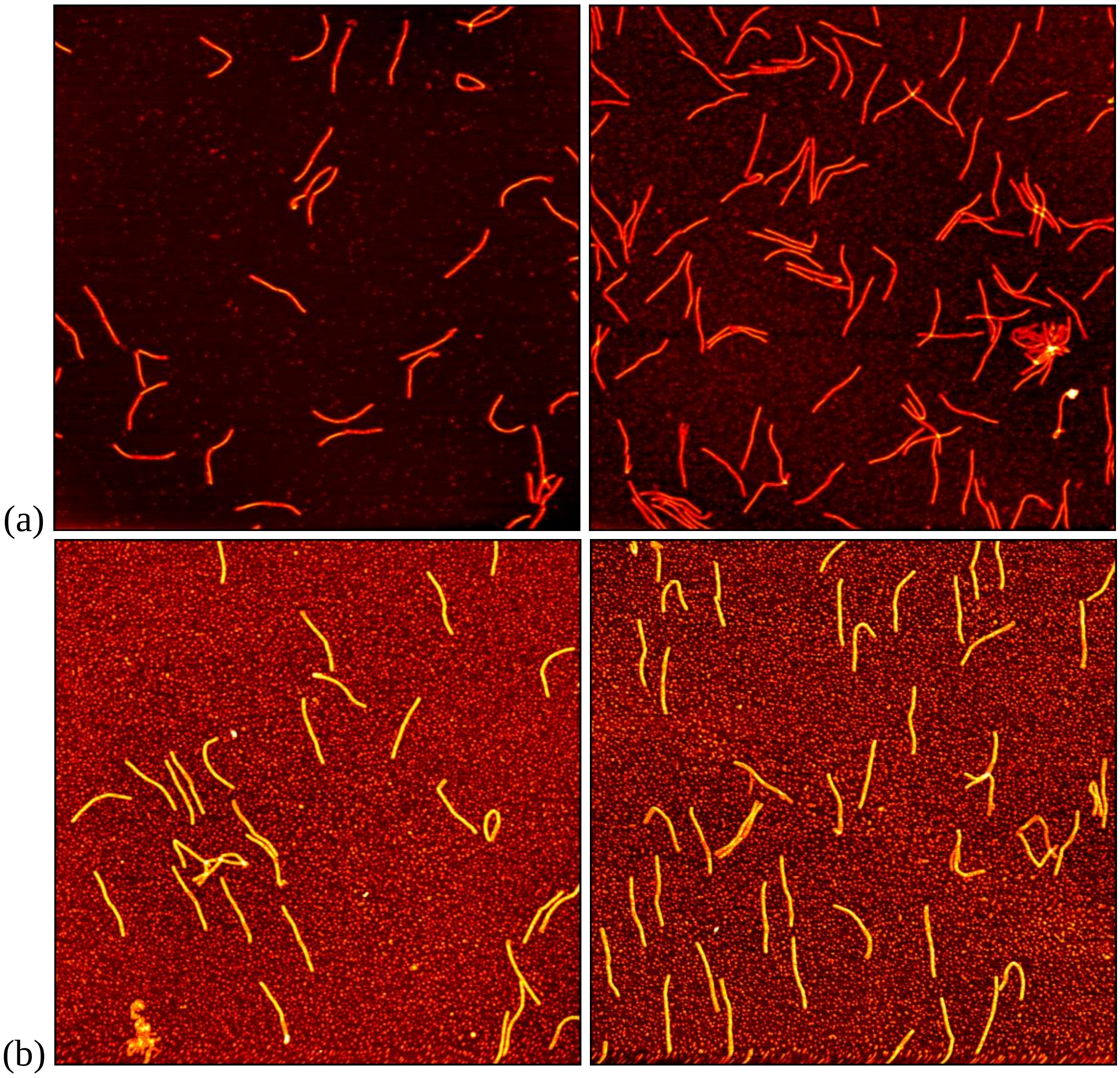

Figure S12: *Additional AFM images of 6HBs adsorbed from PBS (a) and H_2O (b) at the Spdn-modified mica surface. Images are 3 × 3 μm ²and have a height scale of 4 nm.*
